# Assessment of ultrasound ovarian-adnexal reporting & data system (O-RADS) for pediatric patients

**DOI:** 10.1007/s00247-026-06546-w

**Published:** 2026-02-16

**Authors:** Katherine Epstein, Jonathan Dillman, Nadeen Abu Ata, Brian Coley, Yinan Li, Sunny Pitt, Bin Zhang, Rama Ayyala

**Affiliations:** 1 Department of Radiology, Cincinnati Children’s Hospital Medical Center, 3333 Burnett Avenue, Cincinnati, OH 45229 USA; 2 Department of Radiology, University of Cincinnati Medical Center, Cincinnati, OH USA; 3 Department of Radiology, Advent Health Orlando, Orlando, FL USA

**Keywords:** Child Doppler ultrasound, Ovarian cysts, Ovarian neoplasms

## Abstract

**Background:**

Ovarian-Adnexal Reporting & Data System Ultrasound (O-RADS US) is a validated scoring system in adult women with adnexal lesions to help assess the risk of potential malignancy. Limited data exists for children in whom malignancy is rare.

**Objective:**

To evaluate inter-radiologist agreement and diagnostic performance when using the O-RADS US in pediatric patients with ovarian lesions.

**Materials and methods:**

Retrospective IRB-approved study included pelvic ultrasounds (US) from 2015 to 2020 in pediatric patients (<18 years). Pelvic US with ovarian lesions measuring >3 cm in premenarchal patients and >5 cm in menarchal patients were included. Three pediatric radiologists reviewed each US and recorded imaging characteristics and O-RADS classification. Diagnostic performance was assessed, and agreement among radiologists was calculated.

**Results:**

In total, 160 pelvic US exams were included in 160 patients, with a mean patient age of 12.1 years (SD=4.9). Most lesions were classified as O-RADS 2 (almost certainly benign), and fewer cases as O-RADS 4 (intermediate risk) or O-RADS 5 (high risk). Inter-radiologist agreement for O-RADS category was moderate (*κ*=0.42). Diagnostic performance of US O-RADS demonstrated high sensitivity and NPV (100% for all three reviewers). Specificities were 74-82%, and PPV was low at 5-7% for distinguishing malignant/borderline lesions from benign lesions.

**Conclusions:**

Application of the O-RADS US system in pediatric patients may be challenging due to the low overall malignancy rate. Nevertheless, an O-RADS 2 classification provides meaningful reassurance, reflecting minimal malignancy risk in children. Larger studies are needed to determine the clinical utility of O-RADS US and whether pediatric-specific modifications are required.

**Graphical abstract:**

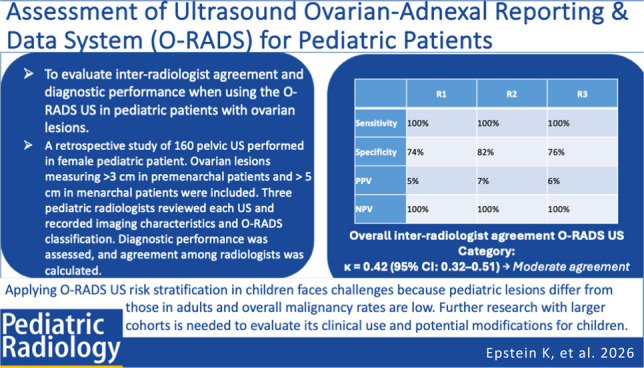

**Supplementary information:**

The online version contains supplementary material available at 10.1007/s00247-026-06546-w.

## Introduction

Ovarian masses are uncommon in the pediatric population, with an incidence of 2.6 per 100,000, and malignant ovarian tumors are even more rare in children [1]. The reported incidence of malignant lesions is 0.102–1.072 per 100,000, with a higher incidence of malignancy among the post-pubertal population (10–19 years of age) [2].

Pelvic ultrasound (US) is the most common first-line imaging modality utilized to assess the ovaries and other pelvic structures in children with abdominopelvic pain, abdominal distension, palpable mass, or precocious puberty. Large size (>8–10 cm) and presence of solid components are findings that favor pediatric ovarian malignancy [3]. Transabdominal ultrasound has high diagnostic accuracy in detecting adnexal pathology in children [4]. Pediatric ovarian malignancies are generally not reliably diagnosed with US imaging alone, as distinguishing benign from malignant lesions can be difficult; however, US serves an important role in the initial assessment and work-up. Prior pediatric US scoring systems, including DePriest and Ueland index scores, have demonstrated high sensitivity and specificity for determining benignity [5]. A more recent risk stratification and standardized lexicon, Ovarian-Adnexal Reporting and Data System (O-RADS), was developed. This system is based on the analysis of International Ovarian Tumor Analysis (IOTA) data that included adult women who were referred for surgery for a known adnexal lesion [6–8]. In adults, ORADS-US demonstrates a high diagnostic performance for ovarian and adnexal lesions (sensitivity and specificity: 84.8–100.8% and 46.4–92.8%, respectively). O-RADS US does not explicitly exclude the use of this system in children; they are implicitly included in the premenopausal group for management stratification. Validation of ORADS US in the pediatric population is limited [9–15]. Further investigation is warranted to determine if ORADS-US can be used for children, and if it requires exclusion criteria or modifications to ensure its appropriate applicability in children.

The purpose of our study is to evaluate inter-radiologist agreement and diagnostic performance when using O-RADS US in pediatric patients with ovarian/adnexal lesions in patients that have undergone clinical pelvic US examinations.

## Material and methods

### Study design

This was a single-center retrospective study that was considered exempt by the local institutional review board at Cincinnati Children's Hospital Medical Center. The requirement for written informed consent was waived. All study activities were compliant with the Health Insurance Portability Accountability Act (HIPAA). No funding was utilized.

Female patients aged 1 day through <18 years who initially presented to and underwent a clinically indicated US examination of the pelvis at our institution between January 2015-December 2020 were identified through a query of our institution’s radiology information system using InSight software (*Softek Illuminate, Overland Park, KS*). US exams that were included in our study had an ovarian/adnexal lesion measuring >3 cm in premenarchal patients (<12 years-old) and >5 cm in menarchal patients ≥12 years-old), based on the clinical radiology report and subsequent review of the report and images to confirm inclusion by a fellowship-trained pediatric radiologist (5 years experience). Menarchal patients were identified from January 2019-December 2020, while a longer time frame (2015-2020) was required to identify a comparable number of premenarchal patients. Menarchal age cutoff was determined by published data indicating an average age for menarche of 12 years [16]. Lesion size thresholds were determined a priori, based on prior literature, to exclude the more common functional and hemorrhagic cysts seen in both cohorts [17]. Exclusion criteria included ovarian/adnexal lesions that did not meet the size criteria as described, and if there was more than one lesion or bilateral lesions. Pathology that was adjacent to but not related to the ovary/adnexa was excluded (such as abscess in the setting of perforated appendicitis).

### Data collection

For each included US examination, deidentified images, including both still images and cine clips, were sent to an external cloud-based server for blinded review (*IntelePACS, Raleigh, NC*). Three board-certified pediatric radiologists at Cincinnati Children's Hospital Medical Center with 6 years, 26 years, and 30 years of post-fellowship experience independently evaluated all cases. They were blinded to patient information, including any other imaging and pathology data, and to each other’s assessments. Before the study, the radiologists received instructions on O-RADS US v2022 and completed a test set of five examinations to ensure its consistent application [6].

Reviewers reported lesion size and laterality. Imaging characteristics evaluated in accordance with O-RADS US v2022 included cystic vs. solid appearing lesion, smooth vs. irregular contours, unilocular vs. bi- or multilocular cystic components, and color Doppler score (numeric categorization of vascularity within a lesion: 1, no flow; 2, minimal flow; 3, moderate flow; 4, very strong flow) (Table 1). Reviewers also recorded if the lesion was a classic benign vs. other lesion. Classic benign lesions include those lesions that can be accurately diagnosed when subjective assessment is made using the O-RADS US lexicon descriptors without concerning features and include typical hemorrhagic cyst, dermoid cyst, endometrioma, paraovarian cyst, peritoneal inclusion cyst, and hydrosalpinx (Table 2). The O-RADS classification score (O-RADS 2–5) was calculated automatically in the data collection system (Research Electronic Data Capture, REDCap) according to published criteria [6–8].

**Table 1:** O-RADS US v2022: assessment categories [6]

O-RADS score	Risk category	Lexicon descriptors
1	Normal ovary	Physiologic cyst: follicle or corpus luteum (≤3 cm)
2	Almost certainly benign (<1%)	- Simple cyst ▪>3 cm - Unilocular, smooth, non-simple cyst ▪>3 cm but <10 cm - Bilocular, smooth cyst ▪>3 cm but <10 cm - Typical benign ovarian lesion ▪<10 cm - Typical benign extraovarian lesion ▪ Any size
3	Low risk (1-<10%)	- Typical benign ovarian lesion ▪≥10 cm - Uni- or bilocular cyst, smooth ▪≥10 cm - Unilocular cyst, irregular ▪ Any size - Multilocular cyst, smooth ▪<10 cm, CS<4 - Solid lesion, ±shadowing, smooth ▪ Any size, CS 1 - Solid lesion, shadowing, smooth ▪ Any size, CS 2–3
4	Intermediate risk (10-<50%)	- Bilocular cyst w/o solid component(s) ▪ Irregular, any size, any CS - Multilocular cyst w/o solid component(s) ▪ Smooth, ≥10 cm, CS<4 ▪ Smooth, any size, CS-4 ▪ Irregular, any size, any CS - Unilocular WITH solid component(s) ▪<4 pps or solid component(s), any size, any CS - Bi- or multilocular cyst WITH solid component(s) ▪ Any size, CS 1–2 - Solid lesion, non-shadowing ▪ Smooth, any size, CS 2–3
5	High risk (≥50%)	- Unilocular cyst ▪≥4 pps, any size, any CS - Bi- or multilocular cyst with solid component(s) ▪ Any size, CS 3–4 - Solid lesion, ±shadowing, smooth ▪ Any size, CS 4 - Solid lesion, irregular ▪ Any size, any CS - Ascites and/or peritoneal nodules

*CS* color score; degree of intralesional vascularity, *pps* papillary projections

**Table 2:** Typical classic benign lesions based on O-RADS US v2022

Lesion	Definition*
Hemorrhagic cyst	Unilocular cyst, no internal vascularity and at least one of the following: • Reticular pattern • Retractile clot
Dermoid cyst	Cystic lesion with ≤3 locules, no internal vascularity, and at least one of the following: • Hyperechoic component(s) with shadowing • Hyperechoic lines and dots • Floating echogenic spherical structures
Endometrioma	Cystic lesion with ≤3 locules, no internal vascularity, homogenous low-level/ground glass echoes, and smooth inner walls/septations(s) •±Peripheral punctate echogenic foci in wall
Paraovarian cyst	Simple cyst separate from the ovary
Peritoneal inclusion cyst	Fluid collection with ovary at margin or suspended within that conforms to adjacent pelvic organs •±Septations
Hydrosalpinx	Anechoic, fluid-filled tubular structure •±Incomplete septation(s) • Endosalpingeal folds (short, round projections around inner walls)

*(6)

Over the study period, the ultrasound systems and corresponding commonly used transducers in clinical practice included Toshiba Aplio XG (6C1,10C3), Toshiba Aplio 500 (6C1, 10C3, 11CI4, 14L5), Canon Aplio i800 (i8C1, 11CI4, 14L5), and Mindray Resona 7 (SC6-1U, SC8-2U, C11-3U). Transducer use was variable based on patient size and age. Imaging protocol for transabdominal pelvic US, when an adnexal or ovarian cyst/lesion was detected, included obtaining longitudinal and transverse images of the lesion with split-screen 2D, including color and spectral Doppler, and longitudinal and transverse images of the lesion with measurements to calculate a volume. All patients in the study cohort had transabdominal ultrasounds, with no transvaginal ultrasound performed in this group.

An electronic health record (*Epic Systems, Verona, WI*) chart review was performed by pediatric radiologist (KE), to document available clinical and imaging follow-up, including follow-up pelvic US(s) and/or MRI. Pathology results were recorded for surgically removed lesions. Patients who had lesion stability on imaging follow-up greater than 2 years or resolution of lesion on follow-up imaging if lesion was not surgically removed were classified as “benign.” Patients without appropriate clinical follow-up or pathology were excluded from the reference standard. The diagnostic performance of O-RADS was assessed using pathology results or appropriate imaging follow-up as the reference standard, as available.

### Statistical analysis

Continuous data were summarized as means and standard deviations; categorial data were summarized as counts and percentages. Fleiss’ kappa statistics (*κ*) were used to characterize inter-rater agreement for O-RADS category and agreement for classical benign vs. other lesion. Agreement based on kappa was interpreted as follows: 0–0.39=poor; 0.40–0.59=moderate, 0.60–0.74=good and 0.75–1.0=excellent [18]. A *P*-value <0.05 was considered statistically significant, and 95% confidence intervals were calculated, as appropriate. Analysis was performed using SAS version 9.4 (*SAS Institute Inc., Cary, NC*).

## Results

### Study sample

**Fig. 1:**
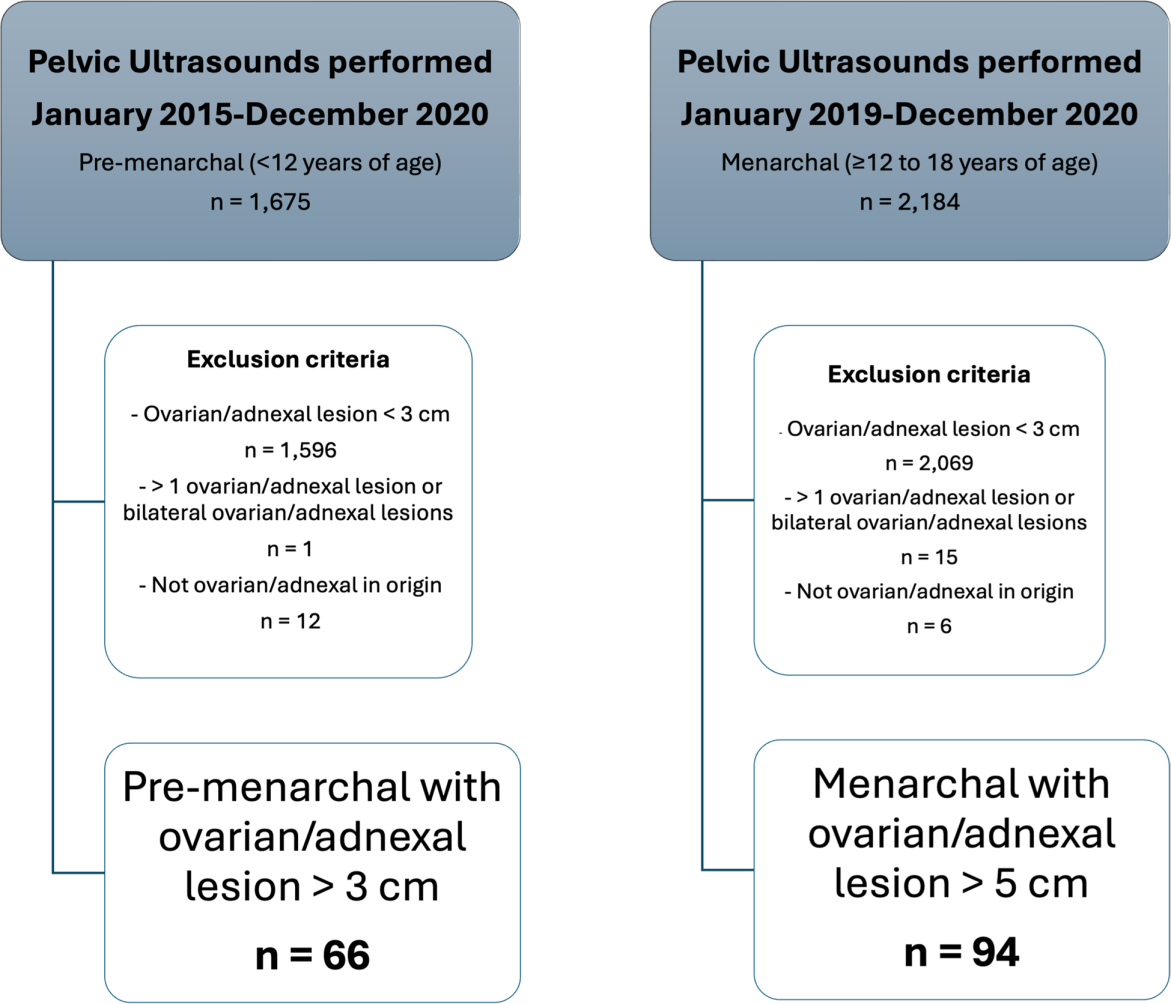
Flowchart demonstrates cohort selection to assess inter-radiologist agreement and diagnostic performance when using the Ovarian-Adnexal Reporting and Data System (O-RADS) US v2022 in pediatric patients with ovarian/adnexal lesions that have undergone clinical pelvic US examinations

Study sample consisted of 160 pelvic US exams performed in 160 patients: 66 cases (34.9%) in premenarchal patients (<12 years of age) and 94 cases (65.1%) in menarchal patients (≥12 years of age). Mean patient age was 12.1 years (SD=4.9; range 1 day-18 years) (Fig. 1).

### Pathology and clinical follow-up

In total, 93.1% (149/160) of patients in our study had clinical and/or imaging follow-up, and 45.0% (67/149) of these patients underwent surgical resection of the lesion. A total of 90.6% (135/149) were benign, 0.7% (1/149) borderline, and 0.7% (1/149) malignant. Of these lesions, 25.5% (38/149) were benign neoplasms. Surgically resected benign neoplasms included serous cystadenoma (*n*=18), mature teratoma (*n*=15), and mucinous cystadenoma (*n*=4). Surgically resected benign nonneoplastic lesions included follicular/paratubal cyst (*n*=16), hemorrhagic cyst (*n*=11), endometrioma (*n*=1). Overall rate of ovarian malignancy in our population was 0.7% (1/149). None of the malignant lesions was within the menarchal cohort (0/94). Both borderline and malignant lesions (serous borderline tumor (11-year-old), and dysgerminoma (10-year-old)) were in the pre-menarchal cohort (2/66 (3%)).

### O-RADS classification

**Fig. 2:**
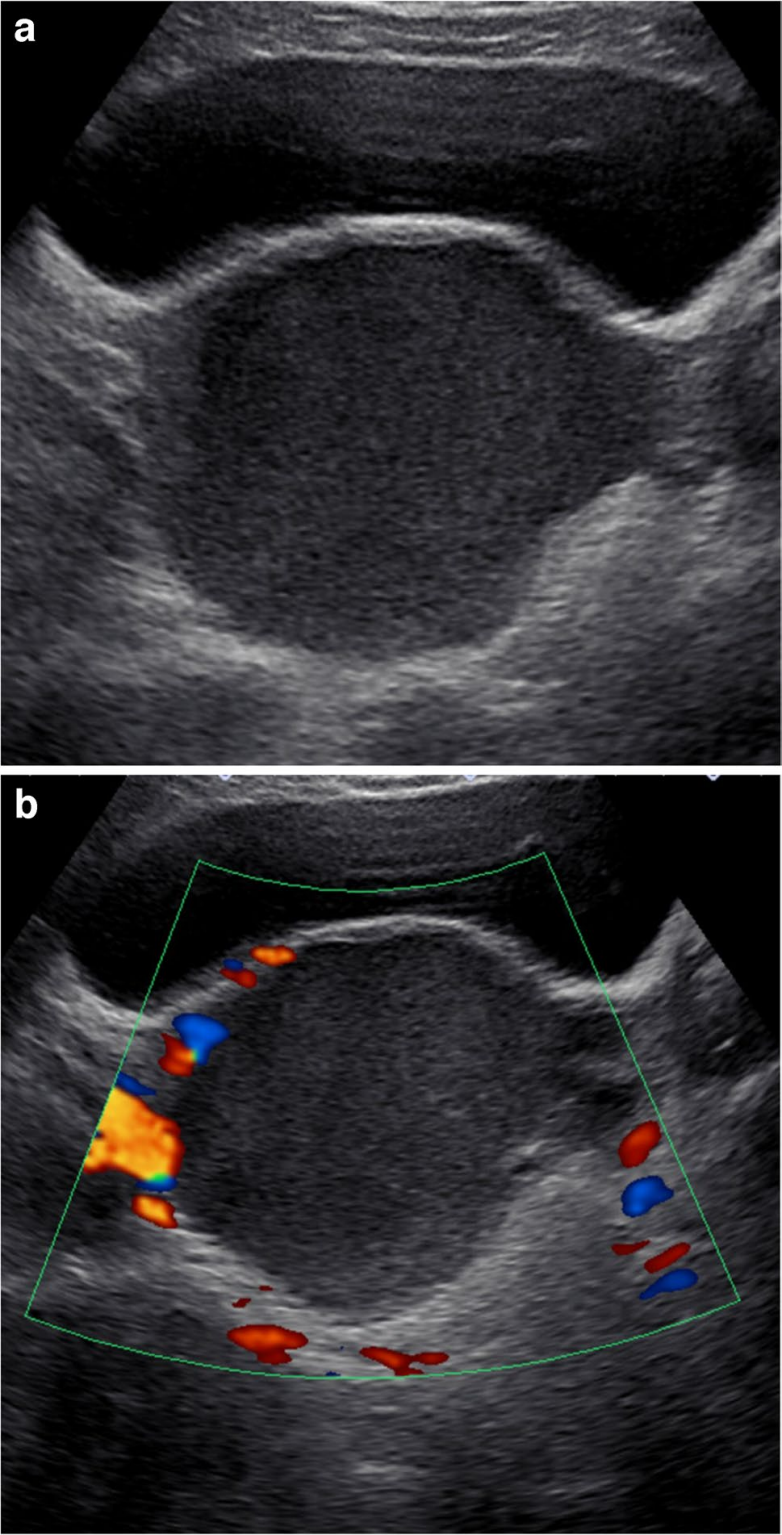
Ultrasound image example of a classic benign lesion, O-RADS 2, which all 3 readers labeled correctly in a 17-year-old female: Transverse static grayscale (**a**) and color Doppler (**b**) ultrasound images demonstrates a 8.9-cm right ovarian cystic lesion with homogenous low-level echoes without internal vascularity. Ultrasound findings are typical for an endometrioma, which was proven pathologically

The majority of lesions were classified as O-RADS 2 (almost certainly benign, <1%: 63.0%/64.8%/67.5% (R1/R2/R3)). Fewer cases were classified as O-RADS 3 (low risk, 1-<10%), O-RADS 4 (intermediate risk, 10-<50%) or O-RADS 5 (high risk, ≥50%): 9.3%/14.8%/7.4%, 25.9%/17.3%/21.6%, 1.8%/3.1%/3.1% (R1/R2/R3), respectively (Table 3). Overall inter-radiologist agreement for O-RADS category was moderate (*κ*=0.42 [95% CI 0.32–0.51.32.51]). Inter-radiologist agreement was poor for classic benign vs. other lesion (*κ*=0.15 [95% CI 0.04–0.26.04.26]), with 84.2%/74.4%/34.7% (R1/R2/R3) lesions recorded as classic benign (O-RADS 2) (Fig. 2). The counts of the features by reviewer for all the cases are listed in Online Resource 1.

**Table 3:** Classification of ORAD category

ORAD category	Premenarchal (*N*=66)			Menarchal (*N*=94)		
	R1	R2	R3	R1	R2	R3
2	38 (57.6%)	36 (54.6%)	37 (56.1%)	62 (66.0%)	67 (71.3%)	71 (75.5%)
3	7 (10.6%)	14 (21.2%)	9 (13.6%)	8 (8.5%)	10 (10.6%)	3 (3.2%)
4	20 (30.3%)	13 (19.7%)	16 (24.2%)	22 (23.4%)	15 (16.0%)	19 (20.2%)
5	1 (1.5%)	3 (4.5%)	4 (6.1%)	2 (2.1%)	2 (2.1%)	1 (1.1%)

O-RADS category distribution was similar between premenarchal and menarchal cohorts (Table 3). The inter-radiologist agreement for O-RADS category was moderate for both the premenarchal group (*κ*=0.38 [95% CI 0.23–0.53.23.53]) and the menarchal group (*κ*=0.44 [95% CI 0.32–0.57.32.57]). Inter-radiologist agreement was poor for classic benign vs. other lesion for both cohorts (*κ*=0.11 [95% CI −0.07 to 0.29] for premenarchal patients, *κ*=0.14 [95% CI 0.01–0.28.01.28] for menarchal patients). The inter-reader agreement and *P*-value for all the features in all cases are included in Table 4.

**Table 4:** Inter-reader agreement and *P*-value for all features in all cases (*N*=160) (*CS*, color score)

	Kappa (95% CI)	*P*-value
Other lesion (cystic or solid)	0.86 (0.70, 1)	<0.001
Benign type (paraovarian or ovarian)	0.79 (0.50, 1)	<0.001
Cystic lesion with or without solid components	0.60 (0.37, 0.83)	<0.001
Smooth or irregular borders	0.56 (−0.12, 1)	0.102
Unilocular or multilocular cystic lesion	0.54 (0.03, 1)	0.042
Multilocular cystic lesion with color score (CS) <4	1 (1, 1)	NA
Lesion size (<10 cm or >10 cm)	1 (1, 1)	NA
Unilocular cystic lesion vs bilocular/multilocular cystic lesion	0.51 (0.11, 0.91)	0.016
Number of papillary projections (<4 or >/=4)	1 (1, 1)	NA
Type of cystic lesion (CS)	1 (1, 1)	NA
Type of solid lesion (CS)	0.37 (−0.47, 1)	0.29

### O-RADS diagnostic performance

**Fig. 3:**
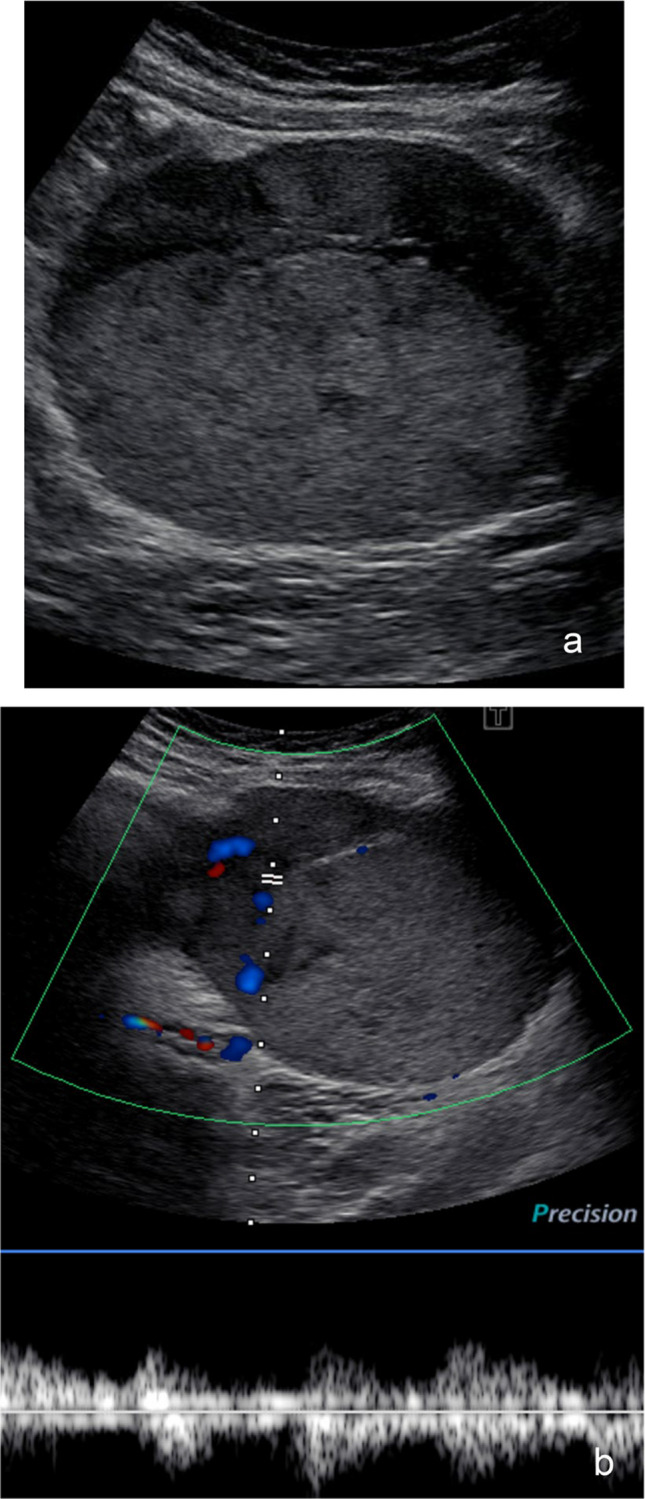
Ultrasound image example of a true positive ovarian/adnexal lesion which all 3 readers labeled correctly in a 10-year-old female: Longitudinal static grayscale (**a**) and color Doppler (**b**) ultrasound images demonstrates a 9.5-cm solid appearing lesion of the right adnexa with smooth outer contour and heterogeneous hyperechoic internal echotexture. Color and spectral Doppler demonstrate vascular flow with low resistance arterial waveforms (color score, CS=2). No shadowing was identified. The three reviewers labeled this lesion as O-RADS 4 (×2) and O-RADS 5 (×1) and was pathologically proven to represent a dysgerminoma

Diagnostic performance of O-RADS US demonstrated high sensitivity and NPV, at 100% for all three reviewers, for distinguishing malignant or borderline lesions from benign lesions (Fig. 3). Specificities for each reviewer were 74%, 82%, and 76%, and PPV was low at 5%, 7%, and 6% (R1/R2/R3).

**Fig. 4:**
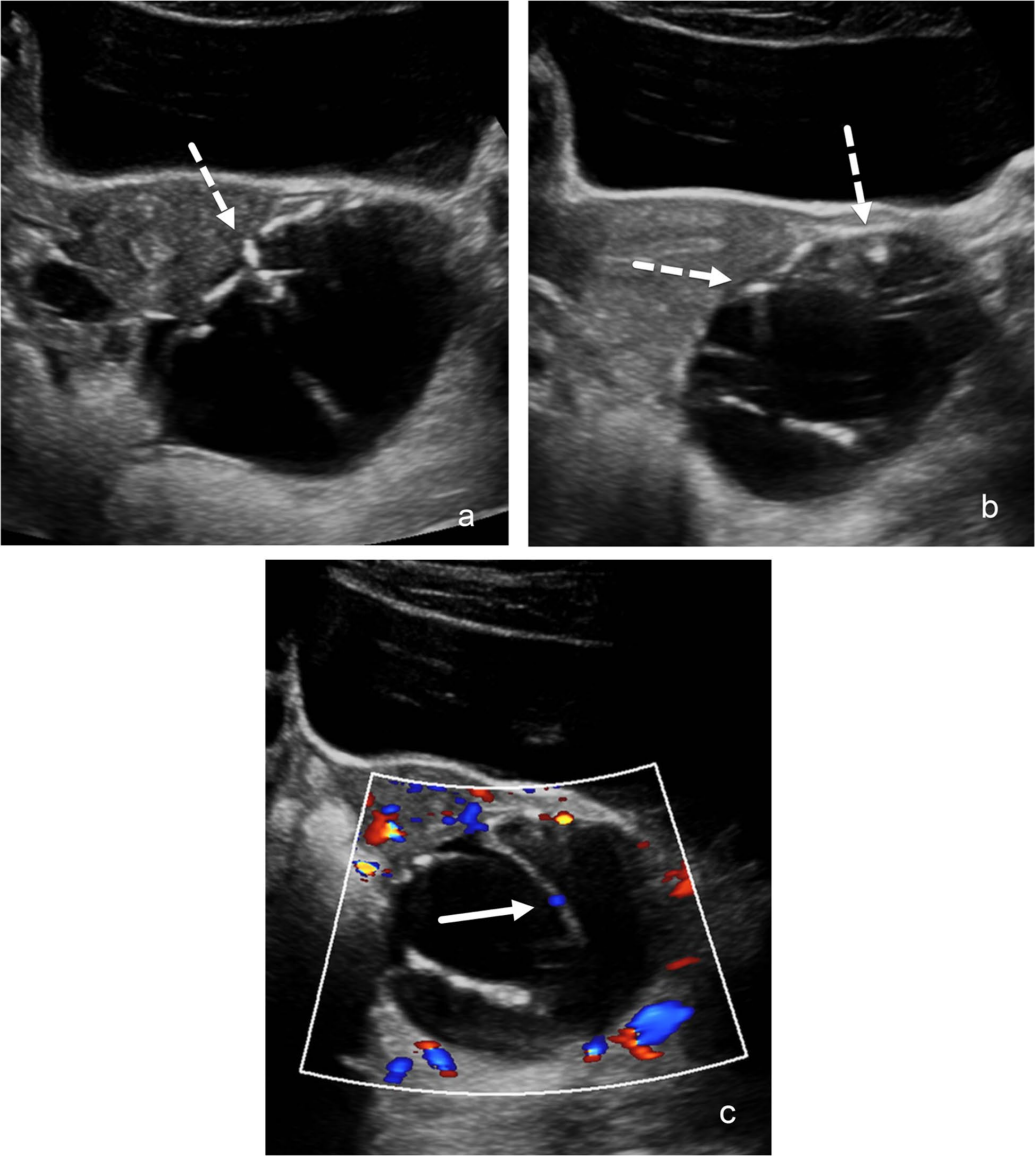
Ultrasound image example of a false positive ovarian/adnexal lesion which all 3 readers labeled incorrectly in a 16-year-old female: Transverse static grayscale (**a**, **b**) and color Doppler **(c**) ultrasound images demonstrates a 7.4-cm complex multilocular cystic lesion of the left ovary, with several internal septations measuring up to 4 mm. Inner walls and septations demonstrate irregular contours, with scattered echogenic foci/calcification (*dashed arrows* in **a** and **b**). No solid components are identified, although there is minimal flow (color score, CS=2) seen on the color Doppler image along a few septations (*solid arrow *in **c**). All three reviewers labeled this lesion as O-RADS 4 that was pathologically proven to represent a mucinous cystadenoma

There was a total of 55 false positive cases (O-RADS 4 and 5) between all three reviewers, which was defined as at least one reader classifying the lesion incorrectly. A total of 47.3% (26/55) of these false positive cases demonstrated agreement with two or more of the reviewers. All of the false positive cases (*n*=55) had either follow-up imaging (*n*=52), pathology (*n*=26), and/or clinical follow-up (*n*=53) which demonstrated the following diagnoses: hemorrhagic or follicular cyst (*n*=34), benign cystic/solid neoplasm (*n*=14), ovarian torsion with associated hemorrhagic/necrotic cyst formation (*n*=5), and Mullerian remnant with hematosalpinx [2] (Fig. 4). The count of the features for all the false positive cases is listed in Table 5.

**Table 5:** Counts of features for false positive cases (*N*=55) by reviewer

	R1	R2	R3
Total number of other lesions	39	45	42
Cystic lesion (±internal echoes, ±incomplete septations):	32	37	33
Solid/solid-appearing lesion (≥80% solid)	7	8	9
Total number benign lesion	16	10	13
Paraovarian (typical paraovarian cyst, typical peritoneal inclusion cyst, typical hydrosalpinx)	0	2	10
Ovarian (typical hemorrhagic cyst, typical dermoid cyst, typical endometrioma)	16	8	3
Type of cystic lesion	32	37	33
(-) Solid component(s)	1	20	7
(+) Solid component(s)	31	17	26
Contours	8	28	16
Smooth	6	18	13
Irregular	2	10	3
Type of cystic lesion with no solid components	1	13	7
Uni- or bilocular	1	11	4
Multilocular	0	2	3
Type of multilocular lesion	0	2	3
CS <4	0	2	3
Lesion size	17	21	10
<10 cm	16	18	10
≥10 cm	1	3	0
Type of cystic lesion	31	24	26
Unilocular	18	15	17
Bilocular or multilocular	13	9	9
Type of unilocular cyst	18	11	17
<4 pps OR solid component that is not a pp	18	11	17
≥4 pps	0	0	0
Type of multilocular lesion	13	6	9
CS 1–2	13	6	9
CS 3–4	0	0	0
Type of solid lesion	5	5	6
Shadowing and CS <4	1	1	1
CS 1	1	3	1
CS 2–3	3	1	4

## Discussion

In our study, there was moderate agreement of O-RADS risk assessment category among the three radiologists who independently reviewed the 160 pelvic ultrasounds. Most adnexal lesions were classified as O-RADS 2 (almost certainly benign). However, a notable proportion of benign lesions were incorrectly categorized as O-RADS 4, i.e., intermediate risk (10-<50%), or O-RADS 5, i.e., high risk (≥50%). This high false-positive rate suggests that the current O-RADS US system may require modification before routine use in pediatric populations to avoid unnecessary anxiety and interventions.

Overall diagnostic performance of US O-RADS in our cohort demonstrated very high sensitivity and NPV (100%), with a specificity ranging from 74% to 82%. In contrast, PPV was very low (5–7%). Therefore, an O-RADS 2 classification, combined with our studies’ demonstration of a very high NPV of the O-RADS classification system, should provide reassurance to both the patient and family that the lesion is benign, with negligible risk of malignancy in the pediatric population.

While O-RADS US has shown a strong diagnostic performance in adult women across multiple validation studies, its application in pediatric populations remains insufficiently validated [6]. Adult studies report sensitivity and specificity ranging from 90.6–98.7% and 81.9–92.8%, respectively, similar to our study and two recent pediatric studies, which reported high sensitivity (99.4%, 100%) and specificity (86.2%, 96.5%) [14, 15]. However, PPV values were much higher in the adult cohorts (70.4% and 83.5%) compared to prior pediatric studies and our study (38.1–45.9%, 5–7%), although one adult study reported a lower PPV of 31.4% [11–13]. Our pediatric study showed a very high NPV, consistent with pediatric and adult data ranging from 98.6% to 100% [7, 14, 15].

Our study had poor inter-radiologist agreement for distinguishing classic benign vs. other lesion (*κ*=0.15) and moderate inter-radiologist agreement for O-RADS category assessment (*κ*=0.42). Prior adult validation studies have assessed inter-radiologist agreement, *κ* values range from 0.31 to 0.77 for O-RADS US category assessment, depending on reader experience and study design; however, they did not directly provide data regarding the agreement for classic benign vs other lesion [11, 19]. Wang and colleagues also reported the inter-observer agreement for the O-RADS category was good (*κ*=0.777) in the pediatric population compared to only moderate agreement in our study (*κ*=0.42). These differences may reflect the study design: Wang and colleagues included only ovarian masses confirmed histopathologically after surgical resection. In contrast, our cohort was obtained via review of consecutive clinical pelvic US exams performed between 2015 and 2020, which is more in alignment with routine clinical practice, where the pretest probability of malignancy is lower than in surgical lesions.

These differences underscore the importance of comprehensive training on O-RADS prior to the imaging review. The training set we provided was limited, therefore future studies would benefit from more cases, including imaging examples of each classic benign lesion and O-RADS US category. Operator dependency in ultrasound may have also contributed to poor inter-radiologist agreement, although our standardized pelvic US protocol aimed to minimize mischaracterization of ovarian lesions. Furthermore, the use of O-RADS US in pediatric population is not common practice and the lack of familiarity of this risk stratification tool in everyday clinical practice likely contributed to the poor agreement.

Additionally, transabdominal imaging was used in our pediatric patients, which deviates from O-RADS recommendations. The governing concepts recommend utilizing transvaginal US to visualize the pelvic organs most effectively, and transabdominal US use is limited to specific scenarios when the transvaginal approach is restricted or cannot be performed [7]. This variation in technique may have impacted both inter-reader agreement and diagnostic performance.

Our findings suggest that O-RADS US is less effective in the pediatric population compared to adults, likely due to the very low incidence of malignant ovarian/adnexal tumors in children. Most ovarian lesions in pediatric patients are benign, such as germ cell tumors or surface epithelial stromal tumors (e.g., mature cystic teratoma, cystadenoma, or mucinous cystadenoma), yet some exhibit sonographic features – such as irregular margins or septations – that mimic higher-risk categories, leading to false positive (O-RADS 4 classification). Continued validation of O-RADS US with larger, multi-center pediatric cohorts (including both academic and non-academic centers) is needed.

Our study has limitations, including being a single institution retrospective study design, which potentially limits generalizability. Many lesions in our study lacked surgical pathology evaluation, although we believe the study design allowed for sufficient follow-up time to determine benignity in those lesions that were not surgically removed. In addition, nearly all the lesions in our pediatric population were benign, resulting in limited true positive cases (ORADS 4 and 5). However, the lesions selected for independent review were consecutive cases presenting to a large pediatric radiology department and were not enriched to include an increased number of malignant cases, which mimics routine clinical practice. The overall rate of malignancy in pediatric ovarian lesions is generally quite low, and our study sample reflects this [2]. O-RADS US was created for use in adult patients, specifically menarchal or post menopausal, while our patient cohort includes pre-menarchal patients. Additional multi-center data would be ideal to further validate the use of the O-RADS US in the pediatric setting, given these differences in patient population, as well as the low incidence of ovarian and adnexal malignancy.

## Conclusions

Our study indicates that applying O-RADS US risk stratification in children presents certain challenges, given that the features of pediatric borderline and malignant lesions differ from those observed in adults, and considering that the overall malignancy rate is low within the pediatric population. Nevertheless, an O-RADS 2 classification provides significant reassurance for both patients and their families, indicating a minimal or insignificant risk of malignancy in the pediatric population. Further research is required involving a larger cohort to assess the practicality of utilizing the O-RADS US system in pediatric clinical settings and to determine whether any modifications are necessary for its appropriate application in this demographic.

## Supplementary information

Below is the link to the electronic supplementary material.

Supplementary file1 (DOCX 4.65 MB)

## Data Availability

No datasets were generated or analysed during the current study.
