# Supplementary material for: Assessment of ultrasound ovarian-adnexal reporting & data system (O-RADS) for pediatric patients
Source: Pediatr Radiol. 2026 Feb 16;56(4):856–66. doi: 10.1007/s00247-026-06546-w (PMC13035678; doi:10.1007/s00247-026-06546-w)
Supplement: Supplementary file 1 [file 247_2026_6546_MOESM1_ESM.docx]

Online Resource 1: Counts of features for all cases (n=160) by reviewer. CS = color score. PPS = papillary projections.

|  | R1 | R2 | R3 |
| --- | --- | --- | --- |
| Total number of other lesions (not benign) | 103 | 121 | 65 |
| Cystic Lesion (+/- internal echoes, +/- incomplete septations) | 91 | 105 | 51 |
| Solid/Solid-appearing Lesion (≥ 80% solid) | 12 | 16 | 14 |
|  |
| Total number benign lesions | 57 | 39 | 95 |
| Paraovarian (Typical Paraovarian Cyst, Typical Peritoneal Inclusion Cyst, Typical Hydrosalpinx) | 9 | 10 | 62 |
| Ovarian (Typical Hemorrhagic Cyst, Typical Dermoid Cyst, Typical Endometrioma) | 48 | 29 | 33 |
|  |
| Type of cystic lesion | 91 | 105 | 51 |
| (-) Solid component(s) | 54 | 81 | 20 |
| (+) Solid component(s) | 37 | 24 | 31 |
|  |
| Contours | 66 | 97 | 34 |
| Smooth | 62 | 85 | 31 |
| Irregular | 4 | 12 | 3 |
|  |
| Type of cystic lesion with no solid components | 52 | 73 | 20 |
| Uni or bilocular | 47 | 68 | 13 |
| Multilocular | 5 | 5 | 7 |
|  |
| Type of multilocular lesion | 5 | 5 | 7 |
| CS<4 | 5 | 5 | 7 |
|  |
| Lesion size | 100 | 102 | 53 |
| <10 cm | 95 | 97 | 52 |
| ≥ 10 cm | 5 | 5 | 1 |
|  |
| Type of cystic lesion | 39 | 32 | 31 |
| Unilocular | 24 | 22 | 21 |
| Bilocular or Multilocular | 15 | 10 | 10 |
|  |
| Type of uniilocular cyst | 22 | 18 | 21 |
| < 4 pps OR solid component that is not a pp | 22 | 17 | 19 |
| ≥ 4 pps |  | 1 | 2 |
|  |
| Type of multilocular lesion | 15 | 7 | 10 |
| CS 1-2 | 14 | 7 | 10 |
| CS 3-4 | 1 |  |  |
|  |
| Type of solid lesion | 10 | 12 | 11 |
| Shadowing and CS < 4 | 3 | 3 | 2 |
| CS 1 | 2 | 8 | 4 |
| CS 2-3 | 5 | 1 | 5 |
